# Somatosensory and Motor Differences between Physically Active Patients with Chronic Low Back Pain and Asymptomatic Individuals

**DOI:** 10.3390/medicina55090524

**Published:** 2019-08-23

**Authors:** Juan Nieto-García, Luis Suso-Martí, Roy La Touche, Mónica Grande-Alonso

**Affiliations:** 1 Departamento de Fisioterapia, Centro Superior de Estudios Universitarios La Salle, Universidad Autónoma de Madrid, 28023 Madrid, Spain; 2 Motion in Brains Research Group, Institute of Neuroscience and Sciences of the Movement (INCIMOV), Centro Superior de Estudios Universitarios La Salle, Universidad Autónoma de Madrid, 28023 Madrid, Spain; 3 Department of Physiotherapy, Universidad CEU Cardenal Herrera, CEU Universities, 46115 Valencia, Spain; 4 Instituto de Neurociencia y Dolor Craneofacial (INDCRAN), 28023 Madrid, Spain; 5 Instituto de Investigación Sanitaria del Hospital Universitario La Paz (IdiPAZ), 28046 Madrid, Spain

**Keywords:** chronic low back pain, psychological variables, sensorimotor variables, pain catastrophizing, fear of movement

## Abstract

*Background and Objectives*: Chronic low back pain (CLBP) is the most common occupational disorder due to its associated disability and high risk of recurrence and chronicity. However, the mechanisms underlying physical and psychological variables in patients with CLBP remain unclear. The main objective of this study was to assess whether there were differences between physically active patients with nonspecific CLBP compared with asymptomatic individuals in sensorimotor and psychological variables. *Materials and Methods*: This was an observational cross-sectional design with a nonprobabilistic sample. The sample was divided into two groups: individuals with nonspecific CLBP (*n* = 30) and asymptomatic individuals as a control (n = 30). The psychological variables assessed were low back disability, fear of movement, pain catastrophizing, and self-efficacy. The sensorimotor variables assessed were two-point discrimination, pressure pain threshold, lumbopelvic stability, lumbar flexion active range of motion, and isometric leg and back strength. *Results*: Statistically significant differences between the groups in terms of catastrophizing levels (*p* = 0.026) and fear of movement (*p* = 0.001) were found, but no statistically significant differences between groups were found in self-efficacy (*p* > 0.05). No statistically significant differences between the groups in any of the sensorimotor variables were found (*p* > 0.05). *Conclusion*: No sensorimotor differences were found between patients with asymptomatic and chronic low back pain, but differences were found in the psychological variables of catastrophizing and fear of movement.

## 1. Introduction

Low back pain (LBP) is the most prevalent musculoskeletal problem and one of the most common reasons for medical consultations [1,2]. Research studies have determined that LBP is the most common occupational disorder due to its associated disability and multifactorial character as well as its high risk of recurrence and chronicity. Chronic LBP (CLBP) not determined by any previous pathology is called nonspecific CLBP and is defined as “…tension, soreness and/or stiffness in the lower back region for which it is not possible to identify a specific cause of the pain. Several structures in the back, including the joints, discs and connective tissues, may contribute to symptoms” [3]. In addition, according to the World Health Organization (WHO), CLBP comprises approximately 85–90% of LBP cases [3].

Previous studies have determined that the chronicity of the symptoms could involve, at the neurophysiological level, a processing alteration at the central level. This alteration contributes to the generation of neuroplastic maladaptive changes at the medullary and the supramedullar levels in which psychological, somatosensory, and motor factors can be affected. [4,5]. It has been observed that patients with nonspecific CLBP present somatosensory alterations, such as a decrease in sensitivity or a lower pain pressure threshold [6]. The presence of tissue hyperalgesia is also common in these patients, suggesting that central sensitization mechanisms are involved [7]. It has been suggested that ongoing nociception is associated with cortical and subcortical reorganization and might play an important role in the process of LBP chronification [8]. Previous studies have suggested that such alterations could be determined by neurochemical changes produced during central sensitization processes in addition to a potential body schema distortion at the cortical level that might lead to alterations in tactile sensitivity [9,10]. These neurophysiological changes at the central level could be related to motor disorders present in patients with CLBP and poorer treatment responses [11]. 

In addition, central sensitization mechanisms could be associated with psychosocial factors, which play a significant role in patients with CLBP. Regarding the influence of psychosocial factors in individuals with nonspecific CLBP, there is an important body of evidence demonstrating that negative psychosocial attributes are strongly associated with a greater perception of pain and disability [12]. Thus, individuals with low levels of self-efficacy have greater fear of movement, which, at the same time, might lead to a greater perception of pain and disability [13,14]. Similarly, it has been observed that negative cognitive factors such as rumination correlate with a lower level of self-efficacy and, together with fear of movement and catastrophism, predict symptom recurrence [12,14,15]. In addition, it has been previously reported that motor and functional factors could be affected, such as extensor muscle strength, which influences motor control, dynamic stability, and range of motion [16]. Along these lines, it has been shown that patients who tend towards fear avoidance behaviors perceive greater disability levels and have less range of motion in lumbar flexion [17]. Similarly, higher levels of pain intensity, anxiety, depression, and fear avoidance result in negative outcomes in physical functioning [18]. Current research findings suggest neurophysiological mechanisms to explain why fear of pain is such a strong predictor of disability [19]. 

In recent years, new lines of research have emerged to identify the mechanisms underlying the associations between physical and psychological variables in patients with nonspecific CLBP [20]. Traditionally, physical inactivity has been associated with the appearance and the onset of CLBP. Previously, it has been shown that physical inactivity is associated with high-intensity low back pain and disability in a dose-dependent manner [21]. Therefore, one of the most commonly used therapeutic approaches in patients with CLBP today is therapeutic exercise and increased levels of physical activity [22]. However, it has been suggested that patients with CLBP are no less active than asymptomatic individuals, and that levels of physical activity are not associated with pain intensity or disability in a direct manner [23,24]. Thus, it remains uncertain which factors beyond activity levels participate in CLBP. Our hypothesis is that physically active patients with CLBP have differences in both psychological and sensorimotor variables related to the onset of CLBP compared with asymptomatic individuals.

Therefore, the main objective of this study was to assess differences between physically active patients with nonspecific CLBP compared with asymptomatic individuals in terms of sensorimotor and psychological variables, with both groups composed of physically active people currently participating in a general exercise program. The secondary objective was to analyze the association between sensorimotor variables and psychological variables in both groups.

## 2. Materials and Methods

### 2.1. Design

An observational cross-sectional study with a nonprobabilistic sample was designed. Participants were allocated into two groups, with the first group composed of patients with nonspecific CLBP, and asymptomatic individuals comprising the control group (CG). The procedures were planned following the ethical considerations of the Helsinki Declaration. The study was approved by the Local Ethics Committee of the La Salle University Centre for Advanced Studies, Madrid, Spain (Project identification code: CSEUL-PI-126/2016; Date: January 2016). This study followed the Strengthening the Reporting of Observational Studies in Epidemiology statement guidelines [25]. All participants completed the informed consent document prior to the study.

### 2.2. Setting and Participants 

A consecutive, nonprobabilistic convenience sample of 30 patients with nonspecific CLBP and 30 asymptomatic individuals for the CG were recruited between April 2017 and July 2017. The sample was recruited from clients of a physical fitness center and patients of a physical therapy clinic, both located in Pozuelo de Alarcón, Spain. 

The inclusion criteria for the CLBP group were (a) low back pain in at least the prior 6 months; (b) low back pain of a nonspecific nature; (c) men and women older than 18 years; and (d) participating in at least 1 moderate exercise session per week. The exclusion criteria were as follows: (a) neurological signs; (b) specific spinal pathology (e.g., inflammatory joint or bone diseases); (c) having had back surgery; and (d) signs of acute inflammation. 

The CG was recruited from among the clients of the previously mentioned fitness center using posters, email newsletters, and social media. Asymptomatic individuals were examined and included in the study if they met the following criteria: (a) individuals who had not had low back pain in the last 6 months; (b) men and women older than 18 years; (c) participating in at least 1 moderate exercise session per week.

There were common exclusion criteria for both groups: (a) cognitive deficiencies that impede correct execution of the tests; (b) illiteracy; (c) understanding or communication difficulties or no comprehension of the Spanish language.

### 2.3. Outcomes

#### 2.3.1. Psychological Variables

##### Low Back Disability

The Spanish version of the Roland Morris Disability Questionnaire (RMDQ) has demonstrated acceptable psychometric properties in measuring the perceived limitation of daily activities as a result of low back pain, and it has a Cronbach’s alpha ranging between 0.84 and 0.93 [26,27]. The RMDQ is a 24-item self-administered questionnaire. Its total score ranges from 0 to 24, where higher scores indicate greater disability [28]. 

##### Fear of Movement

Fear of movement was assessed using the Spanish version of the Tampa Scale for Kinesiophobia (TSK-11), whose reliability and validity have been demonstrated (internal consistency, Cronbach’s alpha = 0.78) [29]. The TSK-11 is a reduced version (11-item) of the TSK-11 questionnaire that eliminates psychometrically poor items from the original version of the TSK-11 and offers the advantage of brevity [30]. Higher scores indicate greater fear of pain and reinjury due to movement.

##### Pain Catastrophizing 

Pain catastrophizing was assessed using the Spanish version of the Pain Catastrophizing Scale (PCS). The PCS has demonstrated good psychometric properties [31]. This scale is composed of 13 items divided into 3 domains: rumination, magnification, and helplessness. Higher scores indicate greater catastrophizing [32].

##### Self-Efficacy

The Spanish version of the Chronic Pain Self-Efficacy Scale (CPSS) has acceptable psychometric properties [33] in assessing perceived self-efficacy (SE) and an ability to cope with the consequences of chronic pain. The CPSS consists of 19 items and 3 domains that assess SE for pain management (PSE), physical function (FSE), and coping with symptoms (SSE). Higher scores indicate greater SE for managing pain [34]. The CPSS presented a reliability of 0.88, 0.87, and 0.90 for PSE, FSE, and SSE, respectively [34].

#### 2.3.2. Sensorimotor Variables

##### Two-Point Discrimination

Research has shown that individual clinicians can reliably assess the 2-point discrimination (TPD) threshold at the neck, the back, the hand, and the foot using mechanical callipers [35]. Participants were positioned in prone decubitus, and the evaluator marked 1 cm lateral of the spinous apophysis of L3 towards the dominant side [36]. The test commenced with callipers set at 70 mm, and the evaluator decreased the distance by 10 mm until the participant was able to perceive only 1 point instead of 2 [35]. This test presented an intraclass correlation coefficient (ICC) of 0.81 (95% confidence interval) [36]. 

##### Pressure Pain Threshold

Algometers are devices that can be used to identify the pressure and/or the force eliciting a pressure pain threshold (PPT). Participants were positioned in prone decubitus, and the evaluator marked at 2 different points: the tibia and the erector spinae muscle. The evaluator then pressed against the tissue vertically, increasing the force at a constant rate of 1 kg/cm^2^. The researcher instructed the individuals to express pain by saying “yes”. This procedure has shown excellent reliability for the low back (ICC 0.86 to 0.99) and moderate to excellent reliability for the tibia (ICC 0.53–0.90). The evaluator took 3 measurements, and the mean was employed in the data analysis [37,38].

##### Lumbopelvic Stability

To assess the ability of the participants to voluntarily control the motion of the lower back, we selected the Hip Flexion Test measured with a “Pressure Biofeedback Unit” (PBU). A validation study concluded that trained examiners can reliably perform PBU measurements for patients with chronic LBP [39]. 

For this test, the participant lay supine with the hips and knees extended, and the PBU was positioned under the lumbar spine so that the inferior border of the device was aligned with the posterior superior iliac spine marks. After the participant was positioned, the PBU was inflated to 40 mm Hg pressure. The participant was then asked to flex their dominant hip, moving the knee towards the chest without touching the table with the foot. Once the maximum hip flexion was achieved, the pressure registered in the PBU was recorded by the assessor. The mean of 3 measurements was used in the analysis.

##### Lumbar Flexion Active Range of Motion

To collect this measurement, a mobile device goniometer called “Goniometer Records” was used. It has shown high intrarater reliability with an ICC greater than 0.80 (95% confidence interval) [40]. It can be used to assess the range of motion of spinal flexion, representing uniplanar movement [41]. 

The clinician held the mobile device over the participant’s sacrum and maintained a slight pressure while the participant performed a “waiter’s bow” movement. The mean of 3 measurements was employed in the data analysis.

##### Isometric Leg and Back Strength

To measure the isometric strength (IS) of the leg and the back extensor muscles, a portable dynamometer was used. The reliability of this device has been validated and can be used for determining leg and back strength in maintained positions, provided that the measurement protocol is standardized [42]. It is a valid and reliable test to measure muscular strength.

For the measurement, the participants let their arms hang straight down to hold the bar with both hands with the palms facing towards the body. The chain was then adjusted so that the knees were bent at approximately 110 degrees. Then, without bending the back, the participant pulled against the weight steadily as much as possible, trying to straighten their legs and keeping the arms straight. The evaluator took 3 measurements, and the mean was employed in the data analysis.

#### 2.3.3. Physical Activity Level

The participants’ levels of physical activity were measured by using the International Physical Activity Questionnaire (IPAQ), which divided the participants into 3 groups according to their level of activity: high, moderate, and low or inactive [43]. This questionnaire has shown acceptable validity and psychometric properties to measure total physical activity.

### 2.4. Procedure 

For this study, both patients with nonspecific CLBP and the CG performed the same procedure following the same instructions. The assessor delivering the physical tests ignored whether the participant performing the test was from the CG or the CLBP group.

Once the participants signed the informed consent document, they received a sociodemographic questionnaire, which collected sex, date of birth, education level, and work situation. In addition, physical activity levels were assessed using the Spanish version of the IPAQ [44]. Once the sociodemographic and the IPAQ questionnaires were completed, the participants completed all 4 psychological questionnaires (RMDQ, PCS, TSK-11, and CPSS) in the same order for all the participants.

Following the completion of the self-reported measures, the participants were instructed to perform the sensorimotor tests in the same order (TPD, PPT, lumbopelvic stability (LS), lumbar flexion active range of motion (LF-AROM) and IS). 

### 2.5. Data Analysis

The sociodemographic and the clinical variables of the participants were analyzed. The data were summarized using frequency counts, descriptive statistics, summary tables, and figures. 

The data analysis was performed using the Statistics Package for Social Sciences (SPSS 20.00, IBM Inc., Armonk, NY, USA). The categorical variables are shown as frequencies and percentages. The quantitative results are represented by descriptive statistics (confidence interval, mean, and standard deviation). For all variables, the z-score was assumed to follow a normal distribution based on the central limit theorem because all the groups had more than 30 participants [45,46]. Student’s *t*-test was used for the group comparisons. Cohen’s *d* effect sizes were calculated for multiple comparisons of the outcome variables. According to Cohen’s method, the magnitude of the effect was classified as small (0.20–0.49), medium (0.50–0.79), or large (0.80). 

The relationships between psychological measures as well as between physical measurements were examined using Pearson’s correlation coefficients. A Pearson’s correlation coefficient greater than 0.60 indicated a strong correlation, a coefficient between 0.30 and 0.60 indicated a moderate correlation, and a coefficient below 0.30 indicated a low or very low correlation [47].

## 3. Results

Table 1 shows the baseline characteristics of the sociodemographic and the activity level variables. The total sample was 60 participants (27 women and 33 men). No statistically significant differences between groups in relation to sociodemographic or activity variables were found (*p* > 0.05) except for weight (*p* = 0.016). There were no statistically significant differences in age, sex, height, education, or physical activity levels (*p* > 0.05). 

### 3.1. Psychological Variables

Table 2 shows statistically significant differences between groups in catastrophizing levels (*p* = 0.026), including helplessness (*p* = 0.036) and magnification (*p* = 0.01) subscales of the PCS questionnaire and fear of movement (*p* = 0.001) in both subscales of the TSK-11 (activity avoidance and harm) (*p* < 0.01). No statistically significant differences between groups were found in any of the subscales of the CPSS, showing high levels of self-efficacy in both groups (*p* > 0.05).

### 3.2. Sensorimotor Variables

Table 3 shows no statistically significant differences between groups in any of the sensorimotor variables (*p* > 0.05). 

### 3.3. Correlation Analysis

Table 4 shows the results of the correlation analysis examining the bivariate relationships among sensorimotor and psychological measures. The strongest correlations found in the analysis were for the CG between fear of movement and LS. Less ability to control lumbopelvic motion was strongly correlated with higher scores on the TSK-11 (r = 0.524; *p* < 0.01). In the CG, a positive correlation was also found between higher FSE and higher LF-AROM (r = 0.406; *p* < 0.05) and low back pressure pain threshold (PPT-LB) (r = 0.412; *p* < 0.05). In the nonspecific CLBP group, a higher SSE was positively correlated with higher PPT both in the tibia (r = 0.382; *p* < 0.05) and the low back (r = 0.415; *p* < 0.05). Finally, a positive correlation between higher scores in the PPT-LB and the RMDQ was found (r = 0.368; *p* < 0.05).

## 4. Discussion

The primary objective of this study was to determine the differences between psychological and sensorimotor measures between two physically active populations, one group with nonspecific CLBP and another group that was asymptomatic. Our results suggest no significant differences in somatosensory and functional variables. There were statistically significant differences in catastrophism and kinesiophobia but not in self-efficacy.

Numerous studies support the influence of various psychological factors in patients with chronic pain conditions. Among the most influential is the fear of movement, which, according to the fear avoidance model, is associated with catastrophic conditions and disability. Thus far, it has been determined that physical activity can act as a mediator, reducing disability and pain and therefore the fear of movement. Previous studies have shown that interventions based on physical activity can lead to changes in the fear of movement variable, despite the fact that the size of the effect has been small to medium. [48,49] On the other hand, other authors show that there are no significant changes in fear avoidance after an active intervention model, but instead in pain and disability. [50,51]. These results might suggest why the active population with nonspecific CLBP presents significant differences in psychological variables compared with asymptomatic individuals. In fact, Marshall et al. (2017) indicated that the best option for an intervention in the psychological factors that are not modified by regular physical activity is probably therapeutic education. [52]. 

Our results showed no significant differences between groups for the psychological variable of self-efficacy or for somatosensory and functional variables. This unusual characteristic of the nonspecific CLBP group can be explained by the socioeconomic context of the participants. Our sample reflects an active population with higher sociocultural and economic levels and patients who did not seek help for their condition. A higher socioeconomic status has been associated with lower disability in patients with CLBP [53]. The sample was recruited from among the clients of a physical activity facility where the individuals participate in two to four supervised exercise classes every week. There has been a considerable increase in the body of evidence showing the beneficial effects of physical activity and exercise in the management of CLBP in terms of certain psychological variables and, above all, on sensorimotor variables, which could justify our results, given both groups performed regular therapeutic exercise every week. [54,55].

According to our findings, patients with various conditions of chronic musculoskeletal pain who seek help present an external locus of control, which could be correlated with a lack of self-efficacy [56]. In addition, it has been shown that lack of self-efficacy correlates with greater pain intensity and greater disability, which is a predictive variable of chronic pain recovery [57,58,59]. This might be one of the reasons why our results did not provide significant differences between groups for the variable of self-efficacy and sensorimotor character, given we had patients with nonspecific CLBP who were not in active search of health care. 

As for the results of the correlation analysis, only one large-magnitude association was found in our analysis. In healthy individuals, the greater the precision in controlling the lumbopelvic movement was, the less fear of movement existed. Physical activity, including motor control exercises, produces hypoalgesic effects [60]. The positive association between movement and psychological wellbeing in patients is key to reducing movement-related fear in patients with chronic pain. It is likely that the sense of stability and control in the lumbopelvic area in those patients facilitates positive behaviors that increase their confidence and reduce their fear of movement.

With respect to the nonspecific CLBP group, the strongest positive correlations were found between PPTs and lumbar disability and between PPTs and coping with symptoms subscales. Along these lines, Immura et al. found a correlation between PPTs in the lumbar region and the level of disability in patients with nonspecific CLBP [61]. In addition, a study performed by Falla et al. determined that the reduction in pain thresholds in patients with LBP was associated with different functional alterations, which suggests it could indirectly have an impact on perceived disability and level of self-efficacy [62]. 

### Limitations

The most important limitation of this study is its cross-sectional design. Our findings are valid for suggesting new hypotheses but not for establishing causal relationships. In addition, no corrections have been made for multiple comparisons, which should be considered in the interpretation of the results. Future longitudinal research is recommended to confirm the hypothesis of this study. Therefore, other sensorimotor measures such as dynamic trunk movement, acceleration, or resistance would be necessary to obtain more consistent conclusions and to confirm these results. It would also be interesting to increase the sample size to confirm whether the obtained results could be extrapolated to larger populations. 

## 5. Conclusions

The results of the present study suggest no sensorimotor differences between asymptomatic and physically active patients with CLBP. However, differences were found in the psychological variables of catastrophizing and fear of movement. No significant correlation was found between sensorimotor and psychological variables except for lumbopelvic stability and less fear of movement in healthy individuals. These results highlight the relevance of psychological variables in patients with CLBP and suggest that these variables should be considered clinically for the management of these patients. 

## Figures and Tables

**Table 1:** Descriptive demographic data and control variables.

	CLBP (*n* = 30)	CG (*n* = 30)	*p*-Value
**Age (years) ^a^**	49.3 ± 11.36	48 ± 10.74	0.65
**Sex ^b^**			0.438
Female (%)	14 (46.7)	13 (43.3)	
Male (%)	16 (53.3)	17 (56.7)	
**Height (cm) ^a^**	172.03 ± 7.55	169.57 ± 6.83	0.19
**Weight (kg) ^a^**	73 ± 12.88	65.07 ± 11.80	0.016
**Educational level ^b^**			0.64
No studies (%)	0 (0)	0 (0)	
Primary education (%)	0 (0)	0 (0)	
Secondary education (%)	3 (10)	2 (6,7)	
College education (%)	27 (90)	28 (93.3)	
**IPAQ ^b^**			0.24
Low level (%)	6 (20)	2 (6.7)	
Moderate level (%)	13 (43.3)	18 (60)	
High level (%)	11 (36.7)	10 (33.3)	

Values are presented as mean ± standard deviation or number (%). CLBP: chronic low back pain; CG: control group; IPAQ: International Physical Activity Questionnaire. ^a^ Independent Student’s *t*-test. ^b^ Chi-squared test.

**Table 2:** Comparative data for the psychological variables (Student’s *t*-test and Cohen’s *d*).

	CLBP (*n* = 30)	CG (*n* = 30)	Mean Difference (95% CI); Effect Size (*d*)
**RMDQ**	4.467 ± 2.97	-	-
**PCS**	12.13 ± 8.5	7.67 ± 6.48	4.47 (0.55 to 8.38) * d = 0.59
**PCS-H**	4.5 ± 2.85	3 ± 2.56	1.5 (0.1 to 2.9) * d = 0.55
**PCS-M**	3.57 ± 2.46	2.07 ± 1.84	1.5 (0.38 to 2.62) * d = 0.69
**PCS-R**	4.07 ± 4.05	2.6 ± 2.7	1.47 (−0.32 to 3.25) d = 0.43
**TSK-11**	25.57 ± 5.65	20.30 ± 5.40	5.27 (2.4 to 8.12) ** d = 0.95
**TSK-AA**	15.67 ± 3.74	12.37 ± 3.42	3.3 (1.45 to 5.15) ** d = 0.92
**TSK-H**	9.9 ± 2.82	7.93 ± 2.86	1.97 (0.49 to 3.44) * d = 0.69
**CPSS**	149.17 ± 21.40	153.4 ± 22.45	−4.23 (−15.57 to 7.10) d = −0.19
**PSE**	33.97 ± 10.70	35.83 ± 13.77	−1.87 (−8.25 to 4.52) d = −0.15
**SSE**	59.77 ± 12.44	61.10 ± 10.85	−1.33 (−7.37 to 4.70) d = −0.11
**FSE**	55.43 ± 5.09	56.47 ± 3.71	−1.03 (−3.34 to 1.27) d = −0.23

CLBP: chronic low back pain; CG: control group. RMDQ: Roland-Morris Disability Questionnaire; PCS: Pain Catastrophizing Scale; PCS-H: helplessness subscale of the PCS; PCS-M: magnification subscale of the PCS; PCS-R: rumination subscale of the PCS; TSK-11: Tampa Scale of Kinesiophobia; TSK-AA: activity avoidance subscale of the TSK-11; TSK-H: harm subscale of the TSK-11; CPSS: Chronic Pain Self-Efficacy Scale; PSE: self-efficacy in pain management; SSE: self-efficacy in coping with symptoms; FSE: self-efficacy in function. * *p*-value < 0.05; ** *p*-value < 0.01.

**Table 3:** Comparative data for the sample of sensorimotor variables (Student’s *t*-test and Cohen’s d).

	CLBP (*n* = 30)	CG (*n* = 30)	Mean Difference (95% CI); Effect Size (*d*)
**PPT-T**	4.95 ± 2.43	5.84 ± 1.9	−0.89 (−2.02 to 0.24) *d* = −0.41
**PPT-LB**	4.62 ± 2.23	5.1 ± 1.94	−0.47 (−1.55 to 0.60) *d* = −0.23
**TPD**	40.6 ± 14.89	38.17 ± 12.24	2.43 (−4.61 to 9.48) *d* = 0.18
**IS**	82.17 ± 35	77.31 ± 28.05	4.86 (−11.55 to 21.27) *d* = 0.15
**LF-AROM**	58.34 ± 14.92	63.94 ± 16.44	−5.60 (−13.71 to 2.51) *d* = −0.36
**LS**	8.76 ± 6.33	6.36 ± 3.15	2.4 (−0.20 to 5) *d* = 0.48

CLBP: chronic low back pain; CG: control group. PPT-T: pressure-pain threshold in tibia; PPT-T: pressure-pain threshold in low back; TDP: 2-point discrimination; IS: isometric strength; LF-AROM: lumbar flexion active range of motion; LS: lumbopelvic stability.

**Table 4:** Pearson correlation analysis.

	PPT-T	PPT-LB	TPD	IS	LF-AROM	LS
**RMDQ**						
**CLBP**	0.329	0.368 *	0.078	−0.228	0.123	0.010
**CG**	−0.312	−0.090	−0.016	−0.260	0.191	0.052
**PCS**						
**CLBP**	0.035	−0.161	0.267	0.019	−0.130	−0.120
**CG**	−0.232	−0.307	0.116	−0.120	−0.268	−0.002
**PCS-H**						
**CLBP**	0.072	−0.133	0.209	0.038	−0.062	−0.181
**CG**	−0.159	−0.223	0.230	−0.036	−0.222	−0.014
**PCS-M**						
**CLBP**	0.004	−0.140	0.245	0.026	−0.257	0.045
**CG**	−0.172	−0.272	0.001	−0.224	−0.309	−0.002
**PCS-R**						
**CLBP**	0.019	−0.159	0.265	−0.002	−0.074	−0.152
**CG**	−0.289	−0.340	0.059	−0.100	−0.223	0.009
**TSK-11**						
**CLBP**	0.037	−0.058	0.335	0.030	−0.194	−0.014
**CG**	−0.128	−0.165	0.199	0.204	−0.207	0.524 **
**TSK-AA**						
**CLBP**	0.015	−0.047	0.301	−0.007	−0.230	0.005
**CG**	−0.107	−0.102	0.204	0.190	−0.187	0.406 *
**TSK-H**						
**CLBP**	0.054	−0.053	0.273	0.069	−0.085	−0.035
**CG**	−0.114	−0.189	0.130	0.157	−0.166	0.502 **
**CPSS**						
**CLBP**	0.221	0.149	−0.061	−0.001	−0.014	−0.076
**CG**	0.044	−0.091	0.135	0.038	0.276	0.030
**PSE**						
**CLBP**	−0.045	−0.175	−0.136	−0.028	−0.175	0.114
**CG**	−0.077	−0.165	0.199	−0.106	0.104	0.040
**SSE**						
**CLBP**	0.382 *	0.415 *	−0.060	−0.044	0.114	−0.224
**CG**	0.077	−0.120	0.072	0.098	0.300	0.059
**FSE**						
**CLBP**	0.091	−0.020	0.178	0.163	0.032	−0.010
**CG**	0.328	0.412 *	−0.132	0.337	0.406 *	−0.138

CLBP: chronic low back pain; CG: control group. RMDQ: Roland-Morris Disability Questionnaire; PCS: Pain Catastrophizing Scale; PCS-H: helplessness subscale of the PCS; PCS-M: magnification subscale of the PCS; PCS-R: rumination subscale of the PCS; TSK-11: Tampa Scale of Kinesiophobia; TSK-AA: activity avoidance subscale of the TSK-11; TSK-H: harm subscale of the TSK-11; CPSS: Chronic Pain Self-Efficacy Scale; PSE: self-efficacy for pain management; SSE: self-efficacy for coping with symptoms; FSE: self-efficacy for function. PPT-T: pressure-pain threshold in tibia; PPT-T: pressure-pain threshold in low back; TDP: 2-point discrimination; IS: isometric strength; LF-AROM: lumbar flexion active range of motion; LS: lumbopelvic stability. * *p* < 0.05. ** p < 0.01.
